# The mRNA component of LNP-mRNA vaccines triggers IFNAR-dependent immune activation which attenuates the adaptive immune response

**DOI:** 10.3389/fimmu.2025.1670350

**Published:** 2025-10-15

**Authors:** Liat Bar-On, Hila Cohen, Uri Elia, Lilach Cherry-Mimran, Ofer Cohen, Noam Erez

**Affiliations:** ^1^ Department of Biochemistry and Molecular Genetics, Israel Institute for Biological Research, Ness-Ziona, Israel; ^2^ Department of Infectious Diseases, Israel Institute for Biological Research, Ness-Ziona, Israel

**Keywords:** LNP-mRNA, innate immunity, type I IFN, IFNAR, adaptive immunity, mRNA, lipid nano particles, vaccine

## Abstract

Encapsulation of mRNA in lipid nanoparticles (LNPs) has established the LNP–mRNA platform as the strategy of choice for the rapid development of vaccines against both existing and emerging pathogens. However, despite its widespread global implementation during the COVID-19 pandemic, the immunological mechanisms underlying its efficacy remain incompletely understood. In this study, we investigated in a murine model, the early and robust innate immunity events elicited following immunization with an LNP–mRNA vaccine. Using mRNAs encoding two different proteins as well as a non-coding sequence, it is demonstrated that the mRNA component—rather than the LNP or the encoded antigen —is essential for inducing a potent innate immune response. This response is characterized by rapid activation of dendritic cells, recruitment of monocytes to draining lymph nodes, and systemic cytokine responses involving activation of various innate immune cell populations. Notably, these effects are all dependent on signaling through the type I interferon receptor (IFNAR). Importantly, we show that even a brief and transient inhibition of IFNAR signaling significantly enhances the ability of the LNP–mRNA vaccine to elicit adaptive immune responses, as evidenced by increased frequencies of antigen-specific CD8^+^ T cells and elevated titers of antigen-specific antibodies. Together, our findings reveal that the strong IFNAR-dependent innate response induced by mRNA can attenuate subsequent adaptive immunity. These insights should be considered in the future design and optimization of LNP–mRNA vaccine platforms.

## Introduction

The development and licensure of mRNA vaccines against SARS-CoV-2 marked the beginning of a new era in vaccinology, offering a safe and versatile platform suitable for rapid mass production. These vaccines have demonstrated high efficacy, eliciting robust adaptive immune responses, characterized by elevated titers of neutralizing antibodies and the induction of antigen-specific CD4^+^ and CD8^+^ T cells (1–7). Despite the widespread use, the fundamental mechanisms by which mRNA confer protection remain incompletely understood.

mRNA vaccine formulations typically comprise two key components: nucleoside-modified mRNA molecules that encode the antigen of interest (8), and lipid nanoparticles (LNP) that encapsulate the mRNA and facilitate efficient delivery of intact mRNA to the cytoplasm of cells which in turn translate the encoded protein (9–11).

Activation of innate immune responses is a critical prerequisite for the effective induction of adaptive immune responses by all vaccines (12, 13). Typically, recognition of pathogen-associated molecular patterns (PAMPs) by pattern recognition receptors (PRRs) leads to the upregulation of co-stimulatory markers and production of cytokines. Together with proper and efficient antigen presentation these responses drive cellular and humoral immunity. Consequently, many vaccines incorporate adjuvants designed to enhance the innate immune activation. LNP-mRNA vaccines do not require co-administration with exogenous adjuvants, likely due to their ability to activate robust innate immune responses, hence possessing by themselves strong adjuvant activity (14, 15). Several studies in both animal models and humans, have demonstrated that LNP-mRNA vaccines stimulate robust innate immune response leading to production of proinflammatory cytokines, particularly those regulated by type I interferons (IFNs). Accordingly, significant innate immune activation has been observed in humans following administration of SARS-CoV-2 mRNA vaccines (15–18). It was shown that LNP-mRNA vaccination of nonhuman primates (NHP) leads to innate immune activation localized at the injected site as well as in the proximal draining lymph nodes (dLN). In these studies, infiltration and activation of dendritic cells (DCs) and monocytes, as well as release of type I IFN–related cytokines were documented (19, 20). Following BNT162b2 vaccination of mice there was a strong innate immune response characterized by a wave of type I interferon (IFN) responses in the dLN, that culminated within one day after vaccination (21).

Although LNP-mRNA vaccines have been extensively studied over the past decade, the mechanism by which they activate the immune system remain incompletely understood. In particular it is essential to define the specific contribution of each component - LNP and mRNA – to the induction of innate immune responses. Historically, a major obstacle to the use of mRNA as a vaccine platform was its recognition by multiple innate immune sensors such as RIG-I, MDA5, TLR7/8 and others (4, 15, 22). However, while RNA sensing has an instrumental role in bridging innate and adaptive immune responses to viral infections, it can also impede the therapeutic efficacy of mRNA vaccines by suppressing translation of the encoded antigen (14). This limitation was surmounted by replacement of the uridine ribonucleosides with naturally occurring uridine analogs - allowing mRNA to evade detection by most innate immune sensors, thereby reducing inflammatory signals and enhancing translation (8, 23, 24). In parallel, utilization of highly purified mRNA enabled the removal of double stranded RNA (dsRNA) molecules, further mitigating inflammation and improving protein expression (25, 26). As a result, nucleoside-modified and highly purified mRNA is now commonly referred to as “immuno-silent” due to its reduced capacity to elicit innate immunity (14, 22).

While the mRNA component is thought to be minimally immunostimulatory, recent studies have investigated the adjuvant properties of the LNP component. For instance, “empty”-LNPs – formulations lacking mRNA – have been shown to promote maturation and cytokine production in various DC subsets as well as monocytes (27). Notably, empty LNPs were documented to exhibit adjuvant characteristics when co-administered with subunit antigens of hepatitis B, dengue, influenza or SARS-CoV-2 viruses (28–30). It was suggested that ionizable lipids, considered critical component of LNPs, appear to mediate this adjuvant effect in IL-6 dependent manner (30). Although the above-mentioned studies strongly suggested that the LNPs carrier is the essential adjuvant component, its characteristics as an adjuvant to induce type I IFN response following *in vivo* vaccination with LNP-mRNA remains unclear (31). Furthermore, most of the studies supporting the immuno-silent nature of modified mRNA were conducted *in-vitro* using cultured cells (8, 25). Thus, the relative contributions of the nucleoside-modified mRNA vs. LNP components to the overall immune response *in-vivo* requires further investigation.

In this report we investigated the early immune following *in-vivo* immunization with LNP-mRNA vaccines. Our findings demonstrate that the mRNA component, rather than the LNP, is essential for triggering a robust innate immune response. Moreover, we show that this innate immune activation is dependent on type I IFN signaling through the interferon-α/β receptor (IFNAR) and that blockage of this signaling pathway can enhance adaptive immune response to mRNA vaccines.

## Materials and methods

### Animals and ethics

All animal experiments in this study were approved by the Institutional Animal Care and Use Committee (IACUC) of the Israel Institute for Biological Research (IIBR). Experimental procedures were performed under protocols M-12-22, M-31–23 and M-28-24. All mice used in this study were maintained according to the guidelines and regulations for animal experiments at the IIBR. Female C57BL/6J (#000664) and IFNAR^-/-^ (#032045) mice were purchased from the Jackson Laboratory (Bar Harbor, ME). Animals were age matched between groups and at age of 6–8 weeks at commence of experiments.

### LNP-mRNA vaccine preparation and characterization

mRNA constructs were purchased from TriLink (San Diego, CA, USA) and included complete N1-methyl-pseudouridine (m1Ψ) nucleotide substitution. All mRNA constructs were codon optimized for expression in mice and included an initiator methionine and a Kozak consensus sequence (32, 33). mRNA was purified by cellulose purification, as previously described (34). Removal of double stranded RNA (dsRNA) contaminants was confirmed using dot blot, and endotoxin levels (measured by LAL Kinetic-QCL kit from Lonza) were <0.05EU/ml. For encapsulation, ionizable lipid (ALC0315, CAYMAN 34337), Cholesterol (Avanti Polar Lipids), distearoyl-sn-glycero-3-phosphocholine (DSPC, Avanti Polar Lipids), and dimyristoyl-rac-glycero-3-methoxypolyethylene glycol (DMG-PEG, Avanti Polar Lipids) were mixed at a molar ratio of 40:47.5:10.5:2 with absolute ethanol and cellulose-purified mRNA payloads were suspended in citrate buffer (50mm, pH 4.5). To create LNPs, a dual-syringe pump was used to transport the two solutions through the NanoAssemblr^®^ micromixer (Precision Nanosystems, Vancouver, British Columbia, Canada) at a total flow rate of 12ml/min. For empty LNPs preparation, citrate buffer was mixed with lipid formulation using the same mixing parameters as mentioned above. The particles were then transferred into dialysis overnight against PBS. Particles in PBS were analyzed for size and uniformity by dynamic light scattering (DLS) (Stunner^®^ by Unchained Labs). RNA encapsulation efficiency was confirmed using the RiboGreen Assay (ThermoFisher Scientific, Waltham, MA, USA) and surface charge was measured by Zetasizer analyzer (Malvern Panalytical Ltd, UK). LNP-mRNA formulations displayed comparable hydrodynamic size of 63nm ± 3.2 (RBD), 68.47nm ± 0.98 (F1), 69.5nm ± 0.66 (non-coding) and 59.5nm ± 1.6 (empty LNP). Polydispersity index (PDI) was 0.11 ± 0.01 (RBD), 0.2 ± 0.02 (F1), 0.13 ± 0.01 (non-coding) and 0.23 ± 0.01 (empty LNP). Encapsulation efficiency for all preparations was >93%.with zeta potential of -8.8mV ± 0.4.

### Immunization and IFNAR blocking

Vaccines were administered by intramuscular injection of 50μl volume into each hind leg, for a total of 100μl. Mice were immunized with either 5μg LNP-mRNA, an equivalent dose of empty LNP or PBS. For IFNAR blocking, mice were injected intraperitoneally (IP) with 2.5mg anti IFNAR monoclonal antibodies (I-401-100, Leinco Technologies) 24hr prior immunization and 24hr post immunization.

### Deucravacitinib treatment

Deucravacitinib treatment was adapted from previously published studies (35, 36). Briefly, Deucravacitinib (MedKoo Biosciences) was dissolved in DMSO to a concentration of 10mg/ml and stored at -20 °C. For injection, a mixture of PEG-300:Tween-80 (both from Sigma-Aldrich) was prepared at a ratio of 18:1. For each injection, 20μl of DMSO with or without the inhibitor was added to 120μl of the PEG300:Tween-80 solution, mixed and further added to 180μl PBS for a total of 300μl (total dose of 0.6mg Deucravacitinib). Mice were injected IP with Deucravacitinib or vehicle 24hr and 4hr before immunization with LNP-RBD.

### Tissue processing

At designated time points post immunization, mice were euthanized by IP injection of Pentobarbiton (300mg/kg) and organs were collected. Inguinal LNs and spleens were digested with 1mg/ml collagenase D (Roche, Mannheim, Germany) for 30min at 37 °C. The digested organs were further mechanically processed and filtered to generate single cell suspensions, followed by Red Blood Cells lysis (R7757, Merck) for splenic cells. For analysis of cellular responses at 3 weeks post vaccination, spleens were dissociated into single-cell suspensions by GentleMACS (Miltenyi, Bergisch Gladbach, Germany), filtered, separated on 1.084gr/ml Ficoll Paque premium (GE) and washed with medium.

### Flow cytometry

The following mAb clones were used for staining: CD3 (145-2C11), CD8 (53-6.7), CD4 (GK1.5), CD11b (M1/70), MHCII (I-A/I-E), CD11c (N418), mPDCA1 (129c), Ly6C (HK1.4), CD103 (2E7), and IFNγ (XMG1.2). All antibodies were purchased from Thermo Fisher Scientific or Biolegend. All washing steps were done using FACS buffer (PBS + 0.5% FBS + 2nM EDTA). For Live/Dead staining, cells were stained with Aqua Fluorescent Reactive Dye (1:300 dilution; L34965, Invitrogen) and incubated for 30min at 4 °C. All samples were incubated with anti CD16/CD32 blocking antibody prior to extracellular staining. Following staining of extracellular markers, cells were fixed and permeabilized using a Cytofix/Cytoperm kit (BD Biosciences) according to the manufacturer’s instructions prior to intracellular staining. For intracellular cytokine staining (ICS), cells were incubated for 5hr with SARS-CoV-2 S1 peptide pool (130-127, Miltenyi) at final concentration of 2µg/ml in the presence of protein transport inhibitor cocktail (eBioschience) as directed. Samples were acquired on LSRFortessa (BD Biosciences) and analyzed with FlowJo V.10 software (TreeStar, Ashland, OR).

### Cytokines quantification

At 6hr post immunization, blood was collected from the tail vein. Following an incubation period of 30min at room temperature serum was separated by centrifugation and the samples were stored at -20°C until further analysis. Serum levels of IFNα, CXCL10, IL-6 and CCL2 were determines by ELISA kits (IFN-α-MFNAS0, CXCL10-DY466, IL-6-DY406, CCL2-DY479; R&D Systems, USA) according to the manufacturer’s instructions.

### ELISA

Direct anti-RBD ELISA was performed for the detection of RBD-specific IgG antibodies in mouse sera. Nunc MaxiSorp ELISA plates were coated with 2μg/ml of recombinant SARS-CoV-2 RBD in carbonate/bicarbonate solution (C3041, Sigma, Israel) overnight at 4 °C. Plates were blocked with PBST buffer (PBS + 0.05% + 2% BSA) for 60 min at 37 °C. Following blocking and washing with PBST buffer (PBS + 0.05% Tween 20), the plates were incubated with mouse serum for 1hr at 37 °C. Following washing, alkaline phosphatase-conjugated anti-mouse IgG was used (diluted 1:1000) as a secondary antibody (Jackson ImmunoResearch, USA). P-nitrophenyl phosphate substrate (N2770, Sigma, Israel) was added after washing, and the optical density was measured by microplate reader (SpectraMax 190, Molecular Devices, CA).

RBD levels in mice sera was determined using SARS-CoV-2 RBD ELISA Kit (Abcam 289833) according to the manufacturer’s instructions.

### Statistical analysis

Statistical analyses were performed using GraphPad software 8.1.1 (La Jolla, CA). Statistical differences between groups were evaluated by unpaired t tests (two groups) or one-way ANOVA (>2 groups) with Tukey corrected for multiple comparisons where p<0.05 = *, p<0.01 = **, p<0.001 =*** were considered significantly different among groups. All experimental data are presented as the mean ± standard error of the mean (SEM).

## Results

### LNP-mRNA vaccine induces robust innate immune response

**Figure 1:**
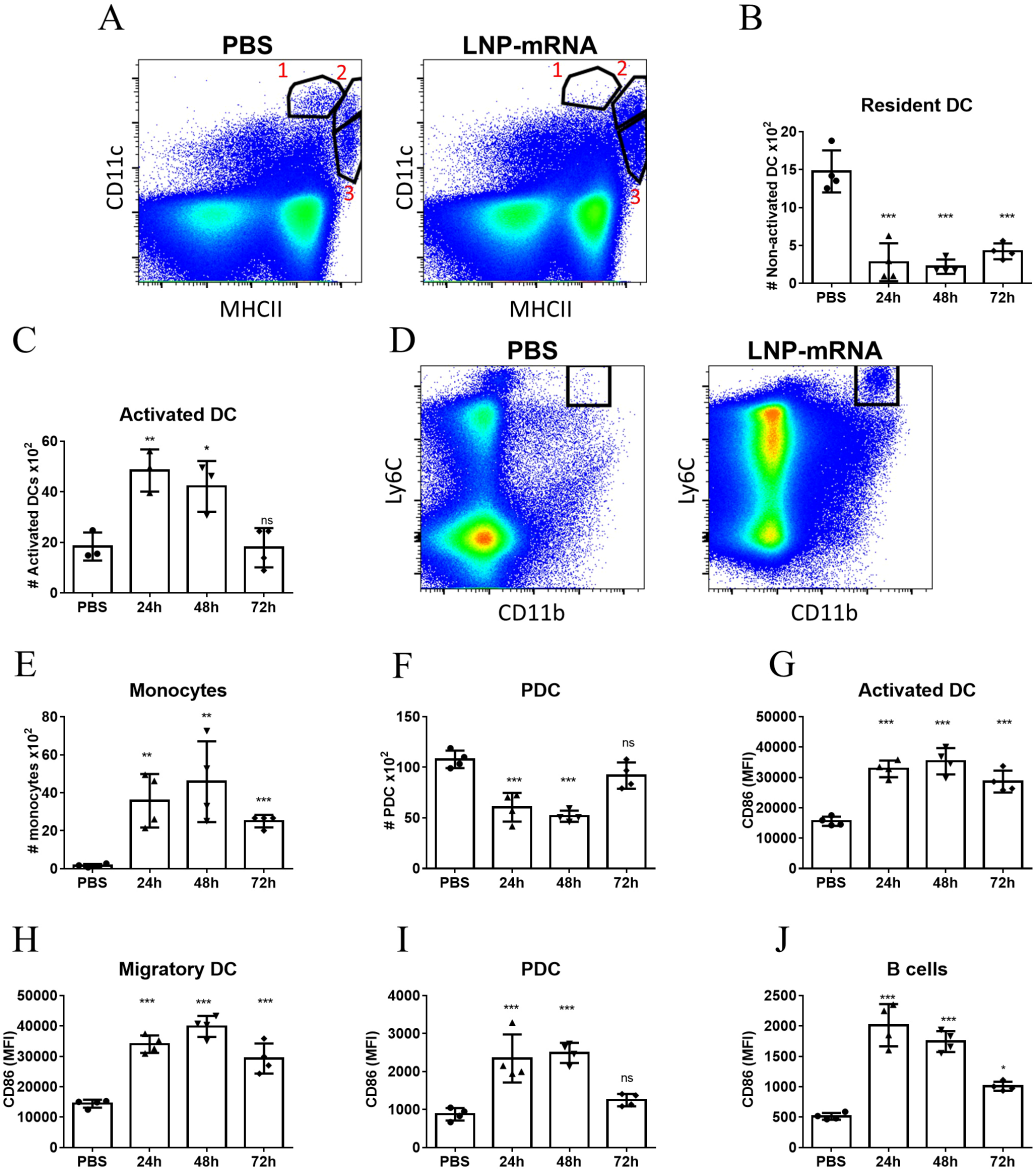
LNP-mRNA vaccine induces robust innate immune response in the dLN. Mice were intramuscularly immunized by i.m injection of LNP-RBD to the hind legs (2.5 µg to each leg). At the indicated time points (24, 48, 72 hours) post vaccination, the inguinal LNs were collected and analyzed by flow cytometry. **(A)** Representative flow-cytometry analysis dissecting the different DC populations in the inguinal dLNs where population 1 represents resident DCs (CD11c^high^MHCII^+^); population 2 represents activated DCs (elevated MHC-II expression); population 3 represents migratory DCs (CD11c^int^MHCII^high^). **(B)** Quantification of resident DCs (population 1). **(C)** Quantification of activated DCs (population 2). **(D)** Representative flow-cytometry analysis of monocytes (CD11b^+^Ly6C^high^) in the dLNs. **(E)** Quantification of monocytes in the dLNs. **(F)** Quantification of PDCs in the dLNs. Activation of different cell populations was also quantified by CD86 expression on activated DCs **(G)**, migratory DCs **(H)**, PDCs **(I)** and B-cells **(J)**. Gating strategy for A-J is presented in **Supplementary Figure S1**. Bars indicate means ± SEM from 3–4 animals per group. Each dot represents a single mouse. Statistical differences were analyzed using one-way ANOVA tests. P values: *P < 0.05, **P < 0.01, ***P < 0.001, and ns, not significant.

It was previously documented that vaccination with a formulation of lipid nanoparticles encapsulating the SARS-CoV-2 spike receptor binding domain (LNP-RBD) elicited antigen-specific T-cells and significant humoral responses which resulted in full protection against challenge with SARS-CoV-2 in a murine model for COVID19 (32). Accordingly, LNP-RBD served in the current study as a model for dissection of the immune response associated with LNP-mRNA vaccination. The effects of LNP-mRNA vaccine on different innate cell populations were initially interrogated in the dLNs by flow cytometry following intramuscular (IM) delivery. At different early time points (24, 48, 72 hours) post vaccination, the inguinal lymph nodes were collected for analysis. At 24hr after vaccination the number of resident dendritic cells (DCs, defined as CD11c^high^MHCII^+^, referred as Population #1 in **Figure 1A**) decreased significantly compared to control unvaccinated mice (**Figures 1A, B**). This reduction was accompanied by significant elevation in the population of activated DCs which was identified by higher MHC-II expression (population #2, **Figure 1A** and **Figure 1C**), implying that resident DCs underwent activation. Notably, the number of migratory DCs (CD11c^int^MHCII^high^, population #3 in **Figure 1A**) in the dLNs was not affected by LNP-mRNA vaccination (**Supplementary Figure S4**). In addition, the number of monocytes (CD11b^+^Ly6C^high^) increased significantly in the dLNs at 24hr (**Figures 1D, E**). This increase sustained for at least 72hr post immunization (**Figure 1E**). The number of plasmacytoid DCs (PDCs, CD11c^low^PDCA1^+^) decreased significantly in the dLNs during the first 48hr and returned to initial levels at 72hr (**Figure 1F**). Moreover, multiple innate immune cell populations including DCs (**Figures 1G, H**), PDCs (**Figure 1I**) and B cells (**Figure 1J**) were highly activated 24hr following vaccination with LNP-mRNA, as indicated by the enhanced expression of the co-stimulation marker CD86. While CD86 levels in both activated and migratory DCs (**Figure 1G** and **Figure 1H**, respectively) remained significantly high in comparison to control animals for at least 72hr post vaccination, they restored to basal level in PDCs and B-cells already after 48hr (**Figures 1I, J**).

**Figure 2:**
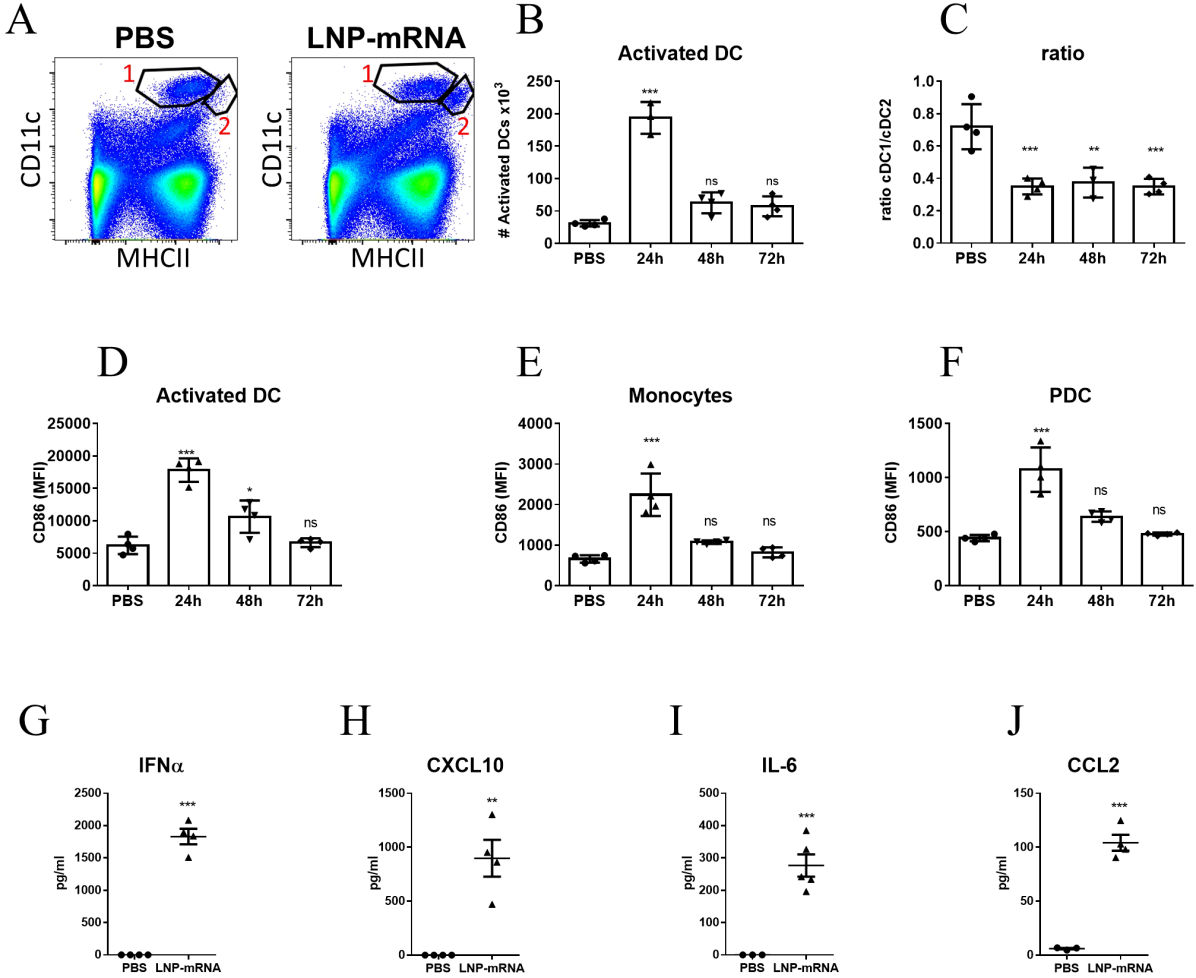
LNP-mRNA vaccine induces systemic innate immune activation. Mice were intramuscularly immunized by i.m. injection of LNP-RBD to the hind legs (2.5 µg to each leg). At the indicated time points (24, 48, 72 hours) post vaccination, the spleens were collected and analyzed by flow cytometry. **(A)** Representative flow-cytometry analysis dissecting the different DC populations in the spleens where population 1 represents DCs (CD11c^high^MHCII^+^) and population 2 represents activated DCs (elevated MHC-II expression). **(B)** Quantification of activated DCs (population 2) in the spleens. **(C)** Ratio between the frequencies of cDC1 (CD8^+^CD11b^-^) and cDC2 (CD8^-^CD11b^+^) sub-populations among splenic DC (population 1). Activation of different cell populations was also quantified by CD86 expression on activated DCs **(D)**, monocytes **(E)** and PDCs **(F)**. Levels of IFNα **(G)**, CXCL10 **(H)**, IL-6 **(I)** and CCL2 **(J)** in the sera of vaccinated mice were measured at 6hr post immunization by ELISA. Gating strategy for A-F is presented in **Supplementary Figure S2**. Bars indicate means ± SEM from 4 animals per group. Each dot represents a single mouse. Statistical differences were determined using one-way ANOVA **(B-F)** or unpaired t tests **(G–J)**. P values: *P < 0.05, **P < 0.01, ***P < 0.001, and ns, not significant.

To address the systemic response, modulations of cell populations in the spleen were examined. In a similar manner to the observations pertaining to dLNs, at 24hr post vaccination, a significant elevation in the number of DCs exhibiting higher levels of MHC-II (population #2 in **Figure 2A**) was recorded (**Figures 2A, B**). In contrast to the prolonged activation of DCs in the dLNs, the elevation in the number of activated DCs in the spleen was transient and returned to the initial base line level as early as 48hr post-vaccination. Moreover, the ratio between the frequencies of CD8^+^ DCs (cDC1 subset) and CD11b^+^ DCs (cDC2 subset) among the non-activated DC population (population #1 in **Figure 2A**) decreased significantly and was maintained low throughout the course of the experiment (**Figure 2C**). As documented above for dLNs, enhanced expression of CD86 was observed in multiple innate immune cells in the spleen including activated DCs, monocytes and pDCs, albeit this activation was transient and lasted during the first 24 hours post vaccination (**Figures 2D–F**). Furthermore, as early as 6 hours post vaccination significant elevation in the levels of IFNα, CXCL10, IL-6 and CCL2 in the serum of vaccinated animals was observed (**Figures 2G–J**). Taken together these data establish that in the current system, LNP-mRNA vaccination induces massive innate immune activation both locally and systemically.

### Innate immune activation by LNP-mRNA is mRNA-dependent

**Figure 3:**
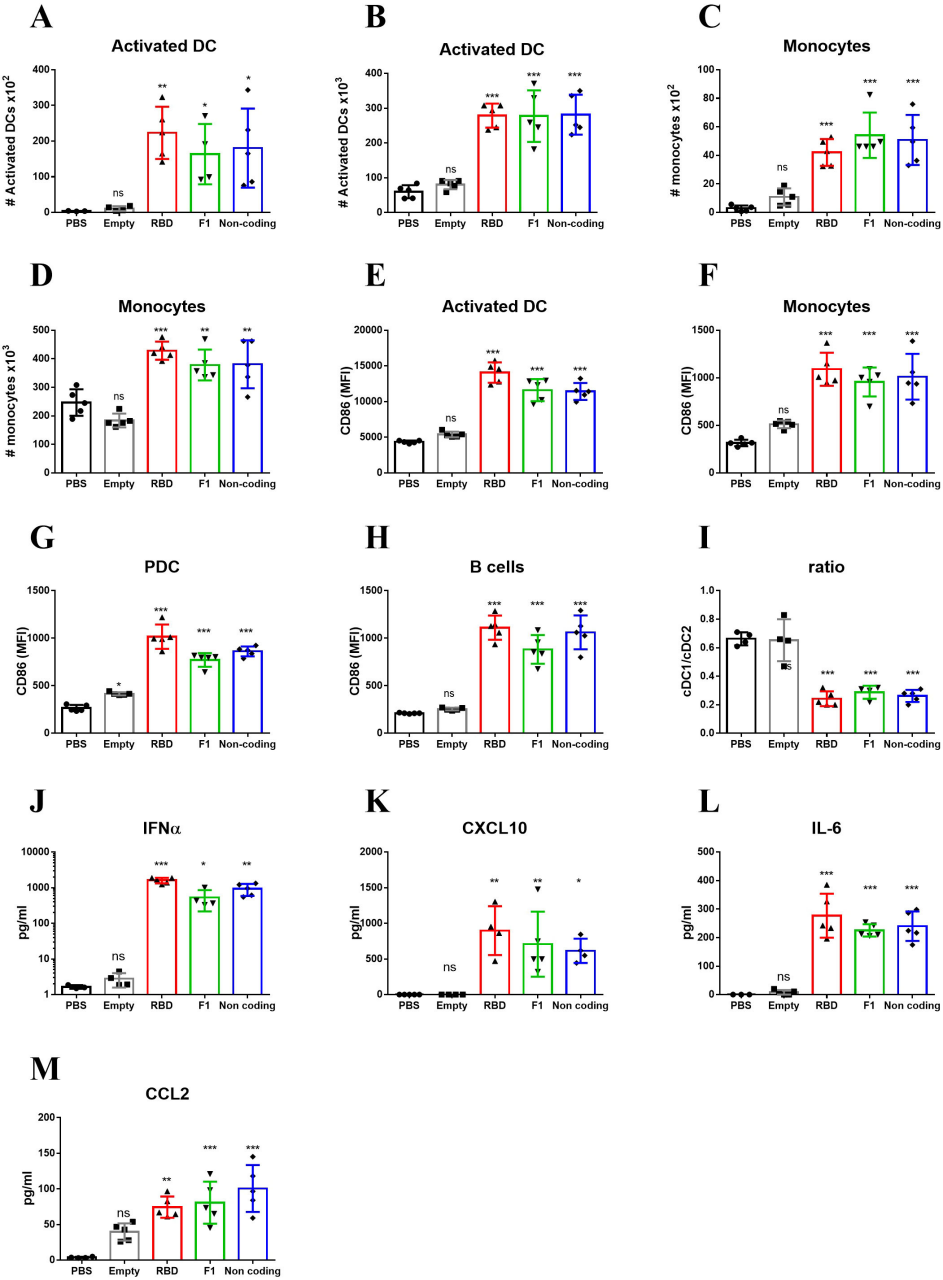
Innate immune activation by LNP-mRNA is mRNA-dependent. Mice were vaccinated with empty LNPs (gray) or LNP-mRNA that encode to either RBD (red), *Y. pestis* F1 (green) or non-coding mRNA (blue). Control group was injected with PBS (black). dLNs and spleen were collected at 24hr post immunization for analyses of various cell populations by flow cytometry. Numbers of activated DCs in the dLNs **(A)** and Spleens **(B)** were quantified as described for **Figures 1**, **2**. Numbers of monocytes in the dLNs **(C)** and Spleens **(D)** were quantified as described for **Figures 1**, **2**. Activation of different cell populations in the spleen was quantified by CD86 expression on activated DCs **(E)**, monocytes **(F)**, PDCs **(G)** and B-cells **(H)**. **(I)** Ratio between cDC1 and cDC2 subpopulations of non-activated DCs (population 1 in **Figure 2**). Levels of IFNα **(J)**, CXCL10 **(K)**, IL-6 **(L)** and CCL2 **(M)** in the sera of vaccinated mice were measured at 6hr post immunization by ELISA. Data are representative of two independent experiments. Bars indicate means ± SEM from 3–5 animals per group. Each dot represents a single mouse. Statistical differences were analyzed using one-way ANOVA. P values: *P < 0.05, **P < 0.01, ***P < 0.001, and ns, not significant.

As explained in the introduction, the rapid activation of the innate immune system following immunization with LNP-RBD, may be the result of response to LNPs, to the mRNA it encapsulates, or to both. Additionally, the magnitude of the response may be affected by the nature of the expressed antigen encoded by the mRNA molecule. To discern among these possibilities, mice were vaccinated with LNP-mRNA that encode to either RBD as described above, *Yersinia-pestis* capsular antigen F1 (33), or non-coding mRNA. Additional control experimental groups were injected with empty LNPs or PBS. Mice were vaccinated and analysis was performed at 24hr post immunization, the time-point of maximal response as described above (**Figure 1** and **Figure 2**). A significant elevation in the numbers of activated DC was observed in both dLNs (**Figure 3A**) and spleens (**Figure 3B**) of mice vaccinated with mRNA-containing particles. It is important to note that the similar increase in activated DCs was observed for both RBD and F1-coding mRNA, as well as for the non-coding molecule. Most notably, empty LNPs did not activate DCs *in-vivo* neither in the dLNs nor in the spleen. These results imply that the presence of the mRNA itself in the LNP-mRNA vaccine is necessary for the robust activation of DCs following vaccination. This inherent ability of mRNA to activate the innate immune system was further confirmed by the rapid recruitment of monocytes to the draining lymph nodes (**Figure 3C**) and spleens (**Figure 3D**) following vaccination with the different mRNA-containing particles, including non-coding mRNA. CD86 expression on DCs, monocytes, PDC and B-cells was similarly elevated solely in the groups of animals immunized with mRNA-containing LNPs (**Figures 3E–H**). In spleens, the described above decrease in cDC1 to cDC2 subsets ratio (**Figure 2C**), was observed only as a result of mRNA presence in the vaccine (**Figure 3I**).

The intrinsic capacity of mRNA to activate the innate immune system *in-vivo* was substantiated by the systemic release of IFNα, CXCL10, IL-6 and CCL2. Thus, similar amounts of these cytokines were induced by all mRNAs including the non-coding mRNA, indicating that the mRNA molecule itself and not the encoded protein is essential for the robust activation of the innate immune system. Of note, empty LNPs did not induce IFNα, CXCL10, IL-6 and CCL2 release, indicating that the activation of innate immune response was specific for mRNA presence (**Figures 3J–M**).

To conclude, the data strongly support the notion that the mRNA component of LNP-mRNA is crucial for the massive innate activation observed early after vaccination. This mRNA-dependent activation was manifested by elevation in MHC-II and CD86 expression, increased numbers of antigen presenting cells and inflammatory cytokines secretion.

### IFNAR signaling pathway is required for the innate immune activation following LNP-mRNA immunization

**Figure 4:**
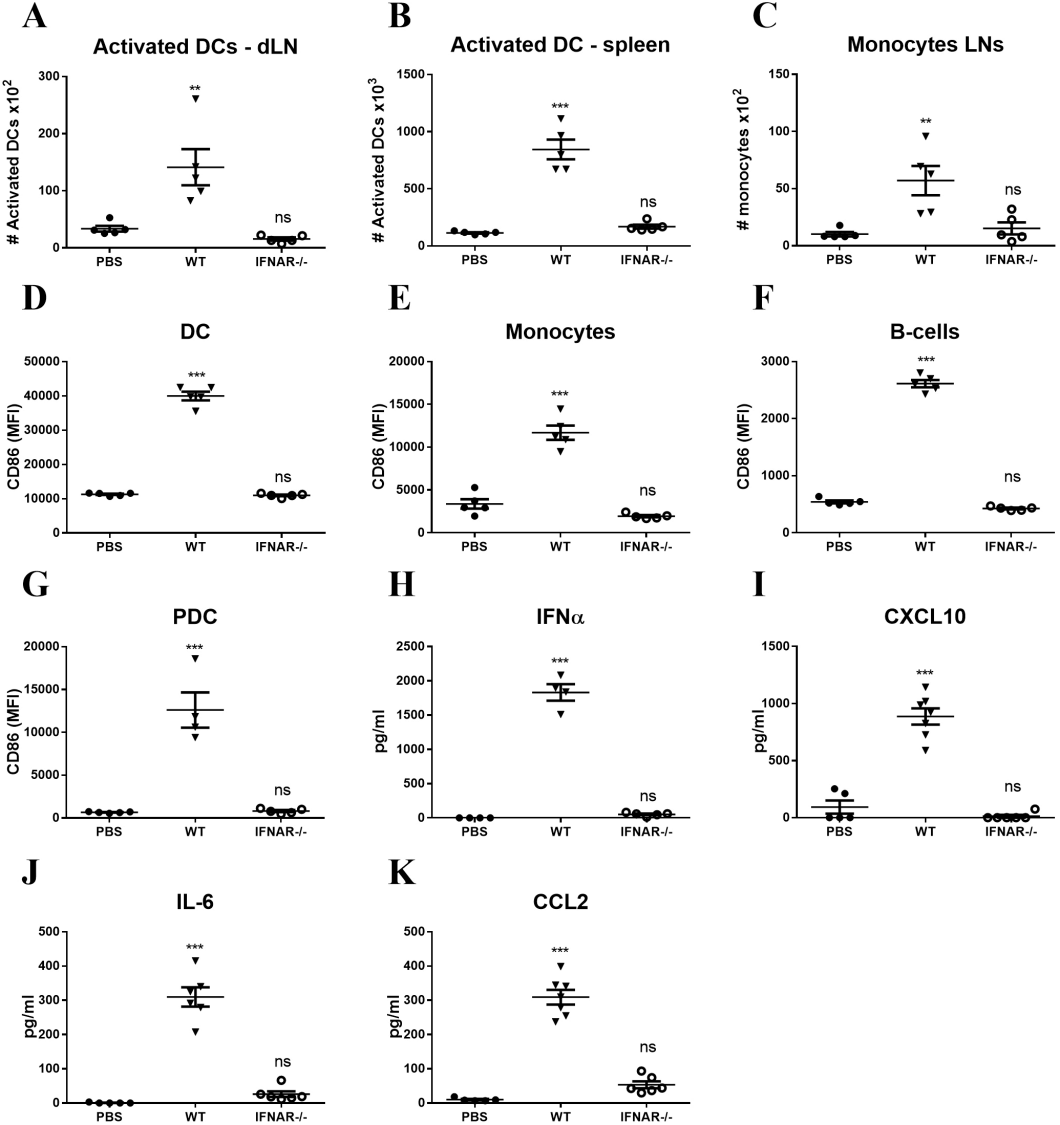
Innate immune activation following LNP-mRNA vaccination is abrogated in IFNAR^-/-^ mice. WT or IFNAR^-/-^ mice were immunized with LNP-mRNA. A group of control WT mice was injected with PBS. dLNs and spleen were collected at 24hr post immunization for quantification of innate immune cell populations by flow cytometry as described in **Figure 1**, **2**. **(A)** Activated DCs in the dLN. **(B)** Activated DCs in the spleen. **(C)** Monocytes in the dLN. Activation of cell populations in the spleen was quantified by CD86 expression on activated DCs **(D)**, monocytes **(E)**, B-cells **(F)** and PDCs **(G)**. Levels of IFNα **(H)**, CXCL10 **(I)**, IL-6 **(J)** and CCL2 **(K)** in the sera of vaccinated mice were measured at 6hr post immunization by ELISA. Bars indicate means ± SEM from 5–7 animals per group. Each dot represents a single mouse. Statistical differences were analyzed using one-way ANOVA. P values: *P < 0.05, **P < 0.01, ***P < 0.001, and ns, not significant.

The immune stimulatory effect of foreign mRNA is known to involve the type I IFN receptor (IFNAR) signaling pathway (37). To determine to what extent IFNAR signaling is required for innate immune activation following LNP-mRNA vaccination, WT or IFNAR^-/-^ mice were vaccinated with LNP-mRNA and the innate immune response was analyzed 24hr later, as shown above. While significant elevation in activated DCs was observed in the dLN and spleen of WT mice, augmentation of activated DCs could not be detected, neither in dLNs nor in spleens of IFNAR^-/-^ mice (**Figures 4A, B**). In a similar manner, there was no elevation in monocyte migration to the dLN in IFNAR^-/-^ mice (**Figure 4C**). In addition, the significant elevation in CD86 expression on different innate immune cells in the spleens and dLNs of WT mice, was not apparent in IFNAR^-/-^ mice (**Figures 4D–G**). Furthermore, in contrast to WT mice, in IFNAR^-/-^ mice no induction of the cytokines IFNα, CXCL10, IL-6 and CCL2 was detected (**Figures 4H–K**). These results strongly suggest that the IFNAR signaling pathway is essential for the mRNA-dependent activation of the innate immune response following LNP-mRNA vaccination. This IFNAR-dependent activation was further characterized to be mediated by the Signal transducer and activator of transcription 1 (STAT1). LNP-mRNA vaccinated mice were pre-treated with Deucravacitinib, an approved TYK2 inhibitor that blocks STAT1/2 signaling (38). In contrast to the robust systemic activation observed following LNP–mRNA vaccination, Deucravacitinib treatment of immunized mice resulted in a marked suppression of the innate immune response. This was evidenced by reduced numbers of activated dendritic cells and decreased expression of the co-stimulatory receptor CD86 across several cell types, including dendritic cells, monocytes, and B-cells(**Supplementary Figure S5**).

### Inhibition of IFNAR signaling enhances the adaptive immune response to LNP-mRNA

**Figure 5:**
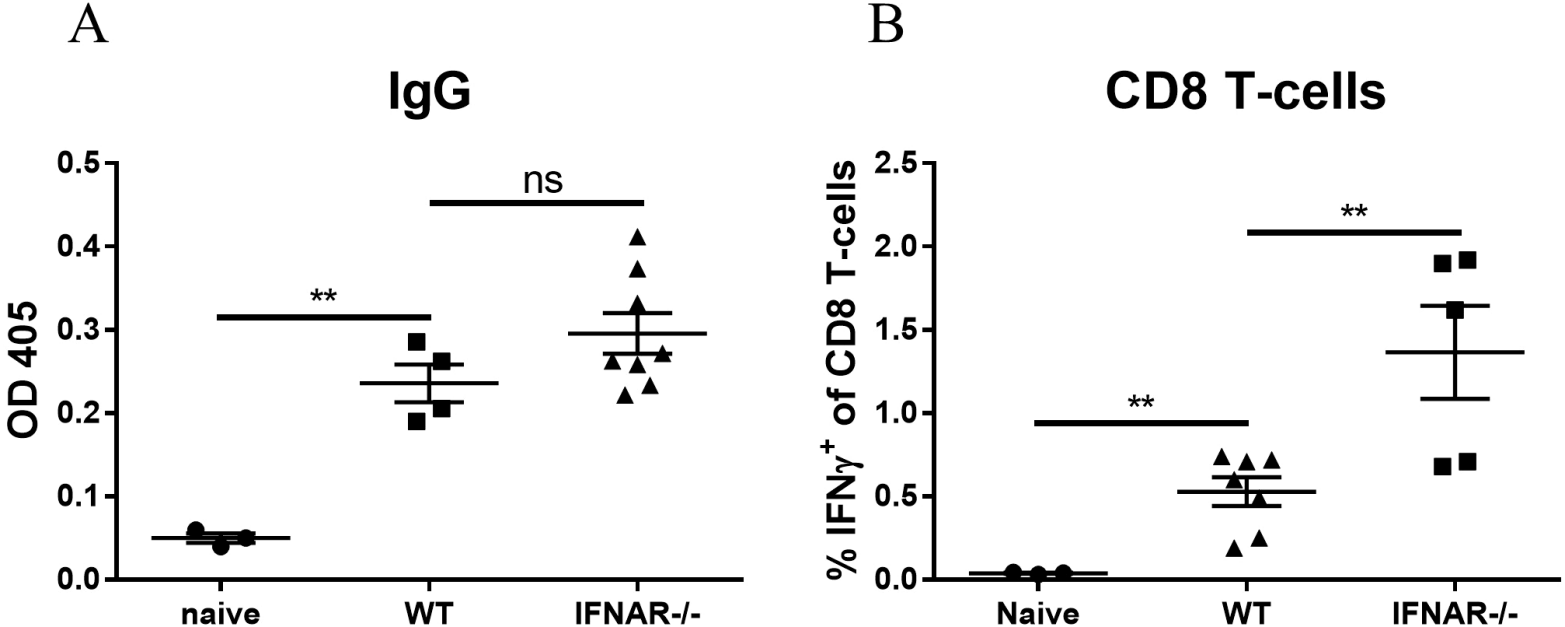
IFNAR deficiency enhances the adaptive immune response to mRNA vaccines. WT or IFNAR^-/-^ mice were immunized with LNP-mRNA. A group of control WT mice was injected with PBS. 3 weeks post vaccination sera and spleens were collected for evaluation of the adaptive immune response. Titers of RBD-specific IgG were determined by ELISA **(A)**. Antigen-specific CD8 T-cells were quantified by ICS for IFNγ **(B)**. Gating strategy for IFNγ positive cells is presented in **Supplementary Figure S3**. Bars indicate means ± SEM from 3–8 animals per group. Each dot represents a single mouse. Statistical differences were analyzed using one-way ANOVA. P values: *P < 0.05, **P < 0.01, ***P < 0.001, and ns, not significant.

Efficient and robust activation of the innate immune response is a pivotal step for an effective adaptive response which further results in the elicitation of antigen-specific humoral as well as T-cell responses. Therefore, we addressed the assumption that the absence of innate activation (observed in IFNAR^-/-^ mice) would affect the induction of adaptive immune responses. At 3 weeks post vaccination, RBD-specific antibody levels in the circulation were measured by ELISA and RBD-specific T cells were quantified by intracellular staining (ICS). Surprisingly, the data evidenced similar titers of specific antibodies in IFNAR-deficient and WT mice (**Figure 5A**) and a significant elevation in the frequencies of RBD-specific CD8-T cells in the spleens of IFNAR^-/-^ mice (**Figure 5B**).

**Figure 6:**
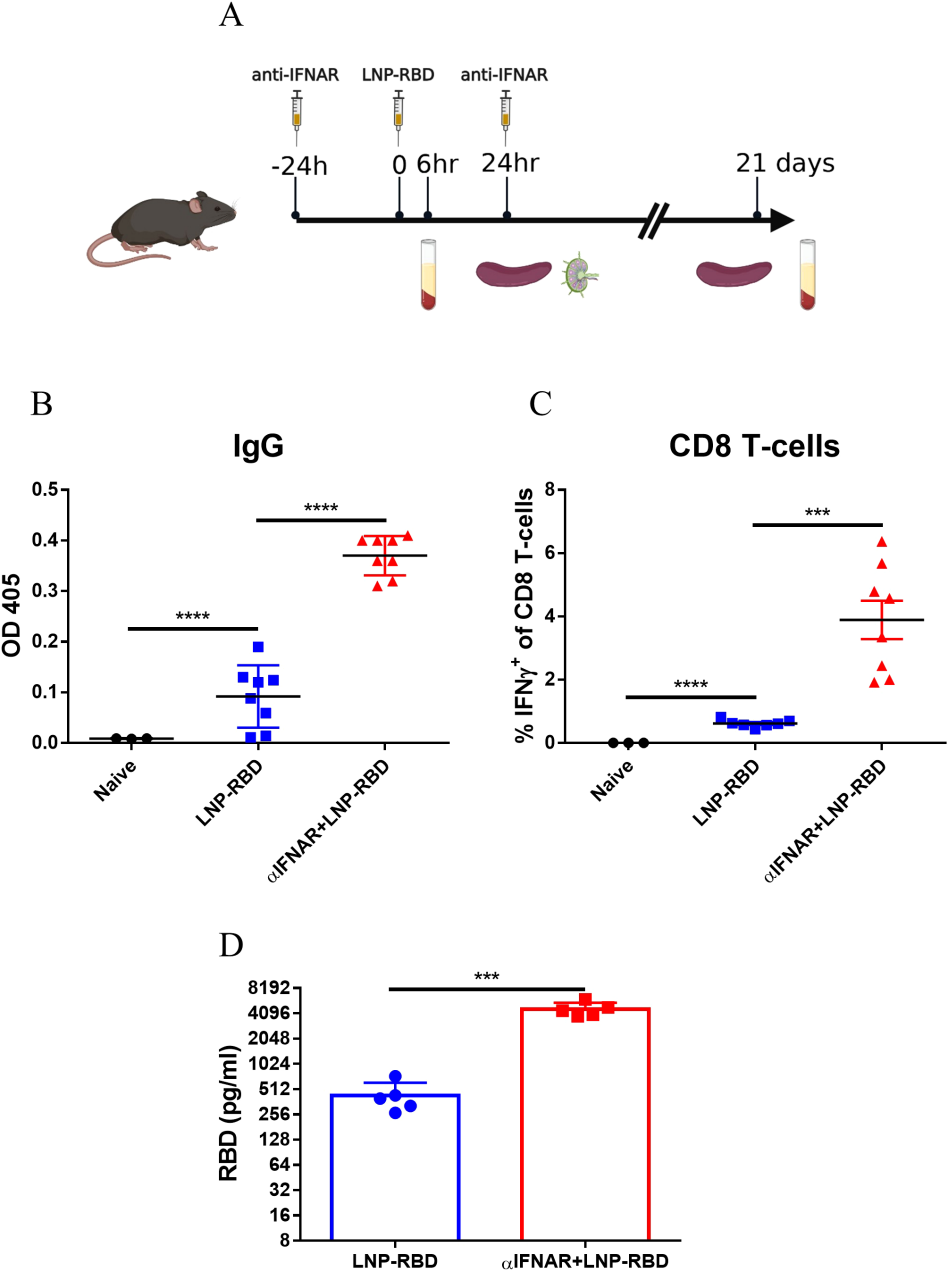
Transient IFNAR blockage enhances the adaptive immune response to mRNA vaccines. Antagonistic IFNAR antibody was administrated 1 day prior- and 1-day post-vaccination. A group of control WT mice was injected with PBS. **(A)** Experimental scheme used in this study for transient IFNAR blocking. 3 weeks post vaccination sera and spleens were collected for evaluation of the adaptive immune response. Titers of RBD-specific IgG were determined by ELISA **(B)**. Antigen-specific CD8 T-cells were quantified by ICS for IFNγ **(C)**. Gating strategy for IFNγ positive cells is presented in **Supplementary Figure S3**. **(D)** 24 hours post vaccination, RBD protein levels in mice sera was determined by ELISA. Bars indicate means ± SEM from 3–8 animals per group. Each dot represents a single mouse. Statistical differences were determined using one-way ANOVA **(B-C)** or unpaired t tests **(D)**. P values: *P < 0.05, **P < 0.01, ***P < 0.001, and ns, not significant.

Complete depletion of IFNAR signaling in IFNAR-/- mice may affect T-cell homeostasis and may possess compensatory signaling pathways affecting adaptive immune response (39–41). Accordingly, an alternative experimental approach was applied, consisting of transient short-term IFNAR-blockage by an antagonistic antibody (**Figure 6A**). Twenty-four hours post vaccination, the immune response was assessed by various parameters, as described above, which established that following transient IFNAR inhibition, both local and systemic innate response, including DC activation, monocyte recruitment, upregulation of co-stimulatory receptors and cytokine secretion were completely abolished (**Supplementary Figure S6**).

The role of IFNAR signaling using the antibody-mediated blockage system, was further used to assess its impact on the development of anti-RBD-specific antibodies and RBD-specific T-cells. Most notably, transient inhibition of the IFNAR signaling pathway resulted in significant elevation both in RBD-specific binding antibodies (**Figure 6B**) and RBD-specific CD8^+^ T-cells (**Figure 6C**), suggesting an unexpected apparent negative effect of type I IFN on the response.

To further investigate the mechanism by which IFNAR blockade enhances antigen-specific T-cell and antibody responses, we hypothesized that inhibition of IFNAR signaling may increase antigen expression from the LNP-RBD vaccine, as previously suggested by others (42). Consistent with this hypothesis, serum RBD levels were elevated approximately tenfold in anti-IFNAR–treated animals compared with vaccinated, untreated controls (**Figure 6D**). These findings indicate that IFNAR signaling strongly suppresses the expression of mRNA-encoded vaccine antigens. When this inhibitory effect is alleviated through IFNAR blockade, antigen expression is markedly increased, thereby driving enhanced induction of antigen-specific T cells and antibodies.

## Discussion

The efficacy of vaccines to elicit protective adaptive immune responses is primarily governed by early events following the encounter of innate immune cells with the vaccine, and the resulting inflammatory environment (12, 13). The world-wide COVID19 vaccination campaign established that LNP-mRNA represents a potent vaccine platform which may be rapidly implemented for emerging disease prevention (1, 2, 4, 5). Yet, in spite of its global use, the immunological mechanisms that governed its efficacy are still elusive.

In the study documented in this report, we first demonstrate that LNP-mRNA vaccination induces a massive innate immune activation both locally and systemically. We show that while the local response in the dLN is present for at least 72hr, the systemic activation of innate immune cells in the spleen is transient and persists for only one day post immunization. Previous studies have shown that mRNA molecules are found in the dLN and in the spleen also, yet to lower extent (21). The lower level of vaccine mRNA in the spleen, can explain the transient activation observed in this organ. The robust modulation of innate immunity in the dLN and spleen was manifested by: (i) systemic elevation in the inflammatory cytokines IFNα, CXCL10, IL-6 and CCL2, (ii) robust activation of DCs, (iii) migration of inflamed monocytes and (iv) elevated expression of the co-stimulation marker CD86 in multiple cells. In addition to these observations, it should be noted that 24hr following vaccination, significant reduction in the number of PDCs in the dLN was detected. This phenomenon is in line with the recent report of Tursi et al. (43) and may suggest that the substantial activation of PDCs resulted in cell death of this population (44). The high levels of IFNα and CXCL10 in the serum, as well as the elevated expression of the co-stimulation marker CD86, indicated a potent type I IFN response early after LNP-mRNA vaccination. Similar innate immune responses and IFNα levels in the serum were documented following immunization of mice with the BNT162b2 COVID19 approved-vaccine (21). Furthermore, elevation in IFNα and pro-inflammatory cytokines was documented in human and Rhesus Macaques following vaccination with BNT162b2 COVID19 approved-vaccine (15–17, 45).

As explained in the Introduction, it is still unclear which components of the LNP-mRNA vaccine are responsible for inducing this massive innate immune response. In the current experimental setup, three different mRNA sequences were used to study the early *in-vivo* events following vaccination. Unexpectedly, immunization with LNP-mRNA coding for either RBD of SARS-CoV-2 spike protein, Y. pestis F1, or a non-coding mRNA resulted in a comparable level of significant activation of the innate immune response. These data demonstrate that the massive innate immune activation is mRNA-dependent and is not affected by protein expression. Most importantly, contrary to the widely accepted notion (14, 30, 46), it is demonstrated here that equivalent doses of **empty LNPs** did not induce any of the evoked responses, suggesting that the presence of the mRNA is essential for the robust innate immune activation following LNP-mRNA vaccination. Our results are supported by previous studies which demonstrated DC activation in the dLN following immunization with LNP-mRNA, but not with empty LNPs (31).

The data showing that immunization with empty LNPs did not induce secretion of IL-6 apparently contradict previous studies (30, 31, 46) which detected IL-6 upon immunization with empty LNPs. This discrepancy may be alleviated by noting that the adjuvant effect of LNPs has been linked to its ionizable lipid component (30, 46) which differed among the referred and current study: the LNP formulation employed by us includes the same ionizable lipid which are incorporated in the BNT162 COVID19 vaccine and differ from the ionizable lipids that were employed by Alameh et al. This may suggest that different ionizable lipids do not share the same potency to induce IL-6 secretion (47).

Thus, our results suggest that the massive innate activation depends on the presence of mRNA. Early studies have established that the use of modified nucleosides and the further mRNA purification to reduce dsRNA, diminish the cellular inflammatory response to the mRNA molecules (8, 24–26). It was therefore considered that the currently used mRNA is immuno-silent (14, 22). Our data suggest that contrary to *in-vitro* studies, when tested *in-vivo*, purified nucleoside-modified mRNA molecules delivered by LNPs are still capable to elicit massive innate immune activation as well as robust type I IFN immune responses.

The current study also shows that the intense innate immune response which is caused by the mRNA component *in-vivo* appears to be totally dependent on the IFNAR signaling pathway. All the above-mentioned parameters of innate immunity including DC activation, CD86 up-regulation, monocytes migration and pro-inflammatory cytokines secretion were completely abrogated in IFNAR^-/-^ mice or following blockage of IFNAR by inhibitory antibodies. The sharp effect of IFNAR-mediated pathway inhibition suggests that no other parallel or compensatory signaling pathways were able to bypass the IFNAR blockade.

Type I IFNs are major regulators of T-cell immunity that can either promote or inhibit T-cell responses (42, 48–51). Furthermore, systemic production of type I IFN has been shown to be crucial to the adjuvant effect of polyI:C as well as other adjuvants (52, 53). Additionally, the innate immune activation characterized by type I IFN responses following LNP-mRNA vaccination is considered to fulfil an important role in the immunogenicity of this platform and its Th1-polarized profile adaptive response (21, 31). Surprisingly, contrary to these concepts, in the current study it is demonstrated that the transient abrogation of the IFNAR signaling pathway which eliminated the innate immune activation, did not attenuate but rather enhanced the efficacy of LNP-mRNA vaccine to induce specific adaptive responses. This was characterized by a significant increase in both antigen-specific CD8 T-cells and antibody titers. Interestingly, in a prime-boost LNP-mRNA vaccination regimen, IFNAR signaling pathway inhibition resulted in CD8 T-cell decrease (21, 31). It may be that Type I IFN can exert stimulatory effect on antigen-experienced rather than naïve T-cells.

A possible mechanism for the observed potentiation of the adaptive response following IFNAR inhibition, may rely on the fact that type I IFNs are potent antiviral cytokines that typically activate RNAses and arrest translation. Accordingly, they may hamper both humoral and cellular immunity by lowering the expression of the antigen encoded by the mRNA (24, 26, 42). Concomitant, we show here that inhibition of Type I interferon dependent signaling, by inhibitory anti-IFNAR antibody resulted in significant increase in RBD level in the serum of LNP-RBD vaccinated mice. Hence, it is noteworthy that in the current study, a short and transient blockage of IFNAR signaling was sufficient to significantly increase protein level encoded by the mRNA vaccine and the subsequent adaptive immune response.

To conclude, this study highlights the contribution of the mRNA component to the massive innate immune activation following LNP-mRNA vaccination and demonstrates that the innate activation has a negative effect on the subsequent adaptive immunity. These findings are important for future design of mRNA vaccines. On a practical note, it may be suggested that attenuating type I IFN induction or IFNAR signaling may improve the potency of LNP-mRNA vaccines.

## Data Availability

The original contributions presented in the study are included in the article/**Supplementary Material**. Further inquiries can be directed to the corresponding author/s.

## Supplementary material

Supplementary Figure 1 Gating strategy to analyze innate immune cell populations in dLNs. Gates of DC subpopulations 1–3 are marked in red.

Supplementary Figure 2 Gating strategy to analyze innate immune cell populations in the spleen. Gates of DC subpopulations 1–2 are marked in red. DCs (subpopulation 1) were further divided to cDC1 and cDC2.

Supplementary Figure 3 Gating strategy to analyze IFNγ producing CD8 T-cells in the spleens.

Supplementary Figure 4 Quantification of migratory DCs (population 3) in the dLNs at the indicated time points (24, 48, 72 hours) post LNP-mRNA i.m. vaccination. Bars indicate means ± SEM from 3–4 animals per group. Each dot represents a single mouse. Statistical differences were analyzed using one-way ANOVA. P values: *P < 0.05, **P < 0.01, ***P < 0.001, and ns, not significant.

Supplementary Figure 5 Activation of immune cell populations following LNP-mRNA vaccination is abrogated following Deucravacitinib treatment. Mice were injected IP with Deucravacitinib or vehicle 24 hours and 4 hours before immunization with LNP-RBD. A group of control WT mice was injected with PBS. Spleens were collected at 24hr post immunization for quantification of innate immune cell populations by flow cytometry. **(A)** Numbers of activated DCs in the spleens as described in **Figure 2**. Activation of cell populations was quantified by CD86 expression on DCs **(B)**, monocytes **(C)** and B-cells **(D**). Bars indicate means ± SEM from 4–5 animals per group. Each dot represents a single mouse. Statistical differences were analyzed using one-way ANOVA. P values: *P < 0.05, **P < 0.01, ***P < 0.001, and ns, not significant.

Supplementary Figure 6 Activation of immune cell populations following LNP-mRNA vaccination is abrogated following transient IFNAR blocking mice. antagonistic IFNAR antibody was administrated 1 day prior and 1-day post- vaccination. A group of control WT mice was injected with PBS. dLNs and spleen were collected at 24hr post immunization for quantification of innate immune cell populations by flow cytometry as described in **Figures 1**, **2**. **(A)** Activated DCs in the dLN. **(B)** Activated DCs in the spleen. **(C)** Monocytes in the dLN. Activation of cell populations was quantified by CD86 expression on DCs **(D)**, monocytes **(E, F)**, B-cells **(G, H)** and PDCs **(I)**. Bars indicate means ± SEM from 5 animals per group. Each dot represents a single mouse. Statistical differences were analyzed using one-way ANOVA. P values: *P < 0.05, **P < 0.01, ***P < 0.001, and ns, not significant.
