# Supplementary material for: The mRNA component of LNP-mRNA vaccines triggers IFNAR-dependent immune activation which attenuates the adaptive immune response
Source: Front Immunol. 2025 Oct 15;16:1670350. doi: 10.3389/fimmu.2025.1670350 (PMC12568647; doi:10.3389/fimmu.2025.1670350)

Supplementary Fig1

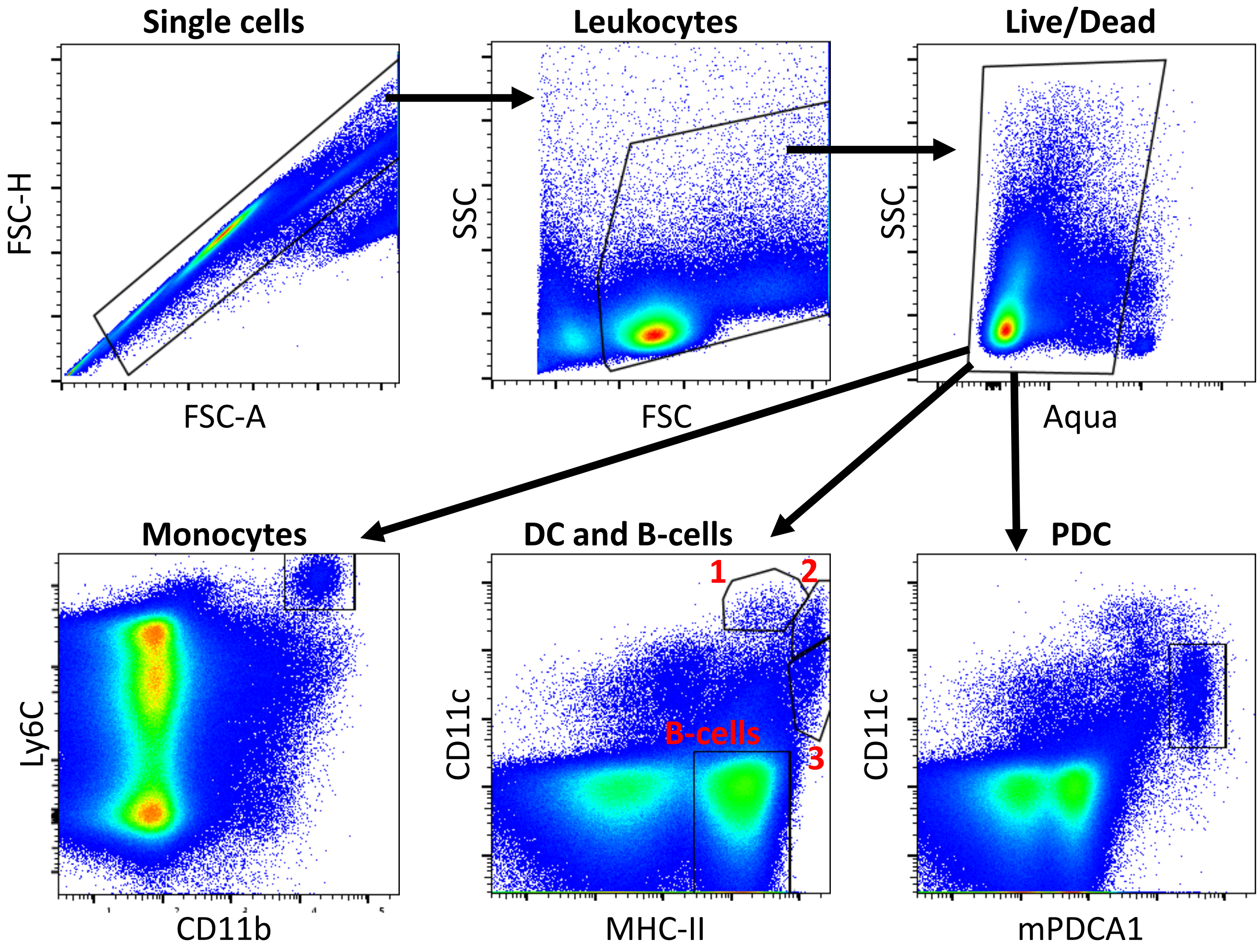

Supplementary Fig2

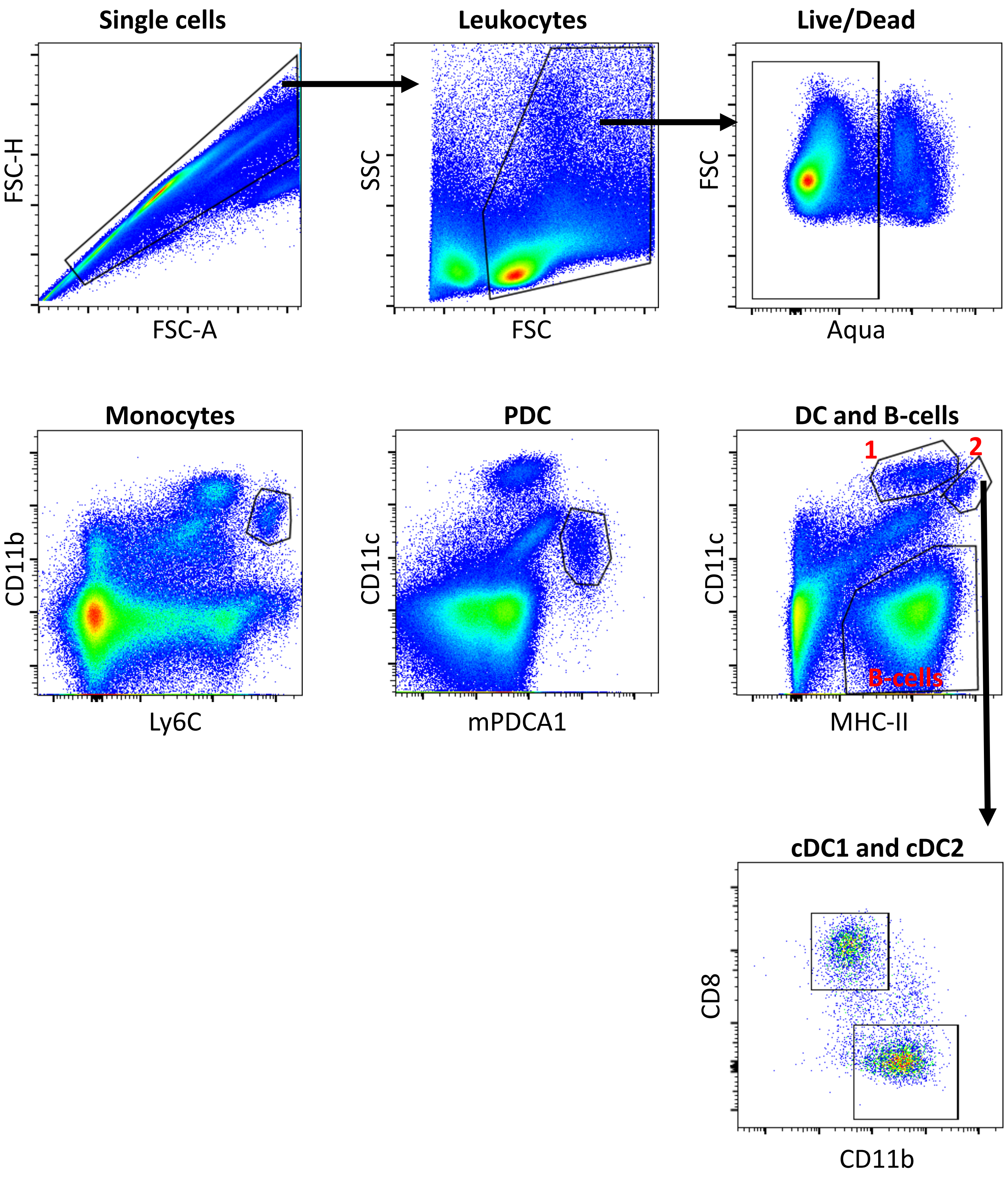

Supplementary Fig3

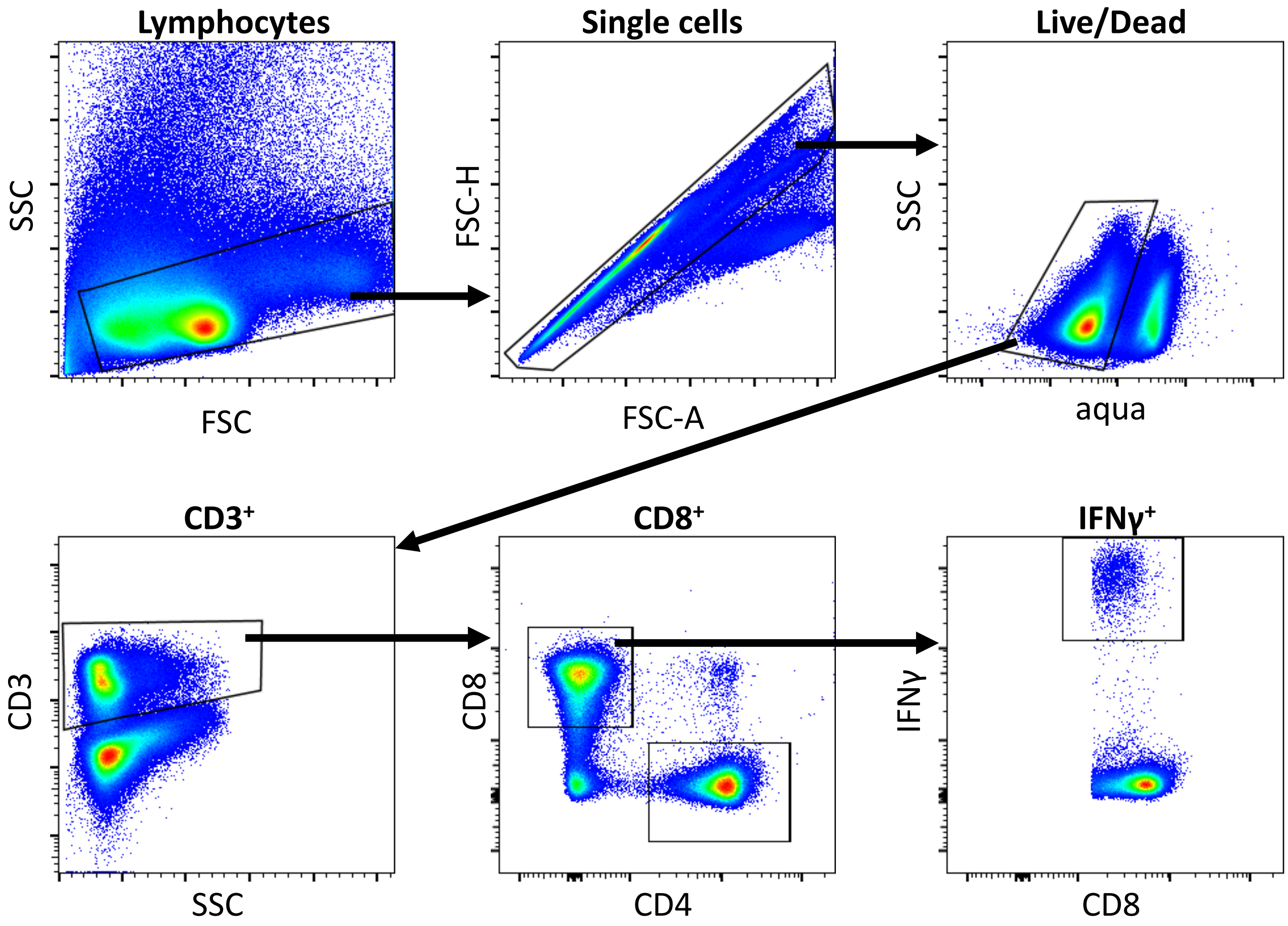

Supplementary Fig4

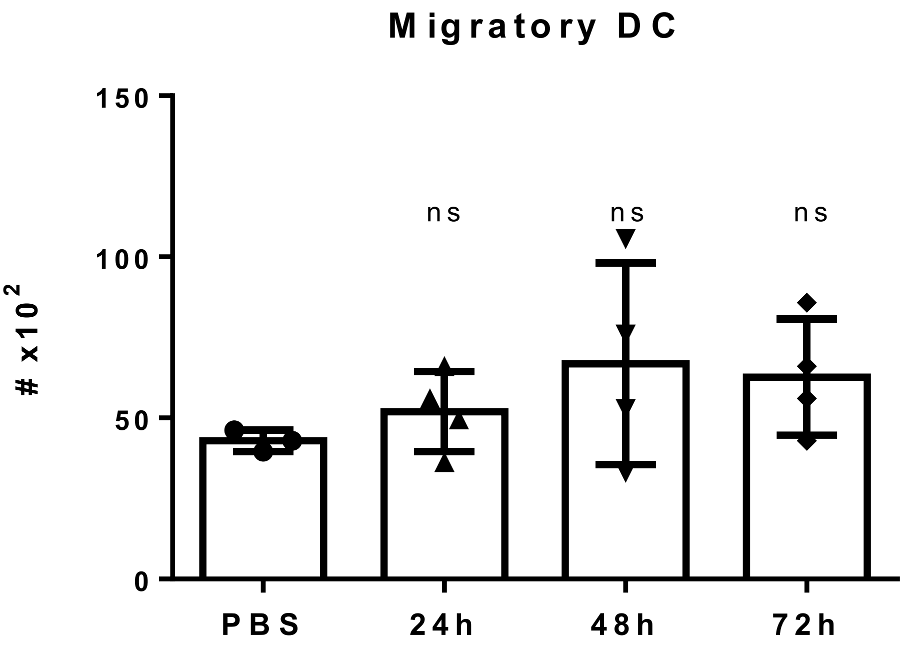

Supplementary Fig5

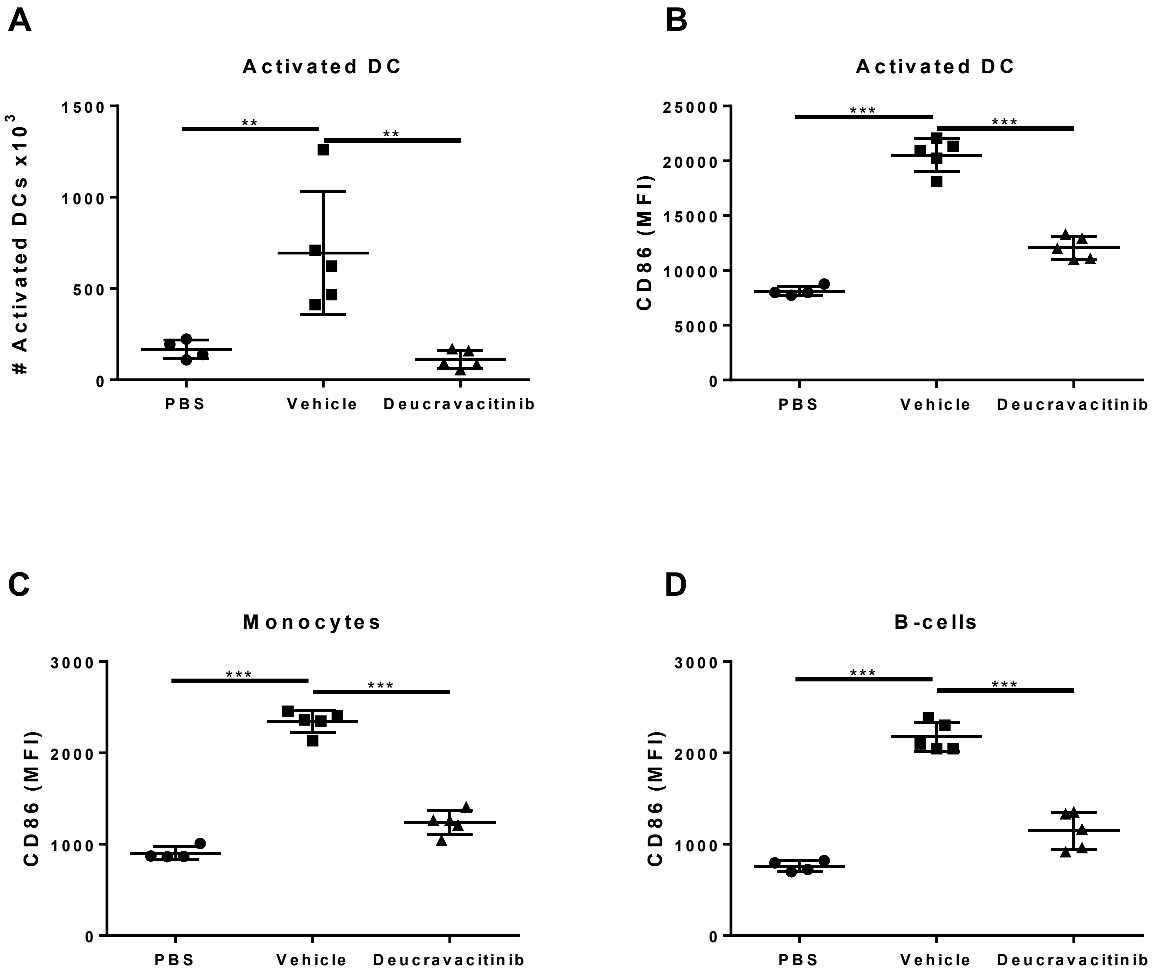

Supplementary Fig6

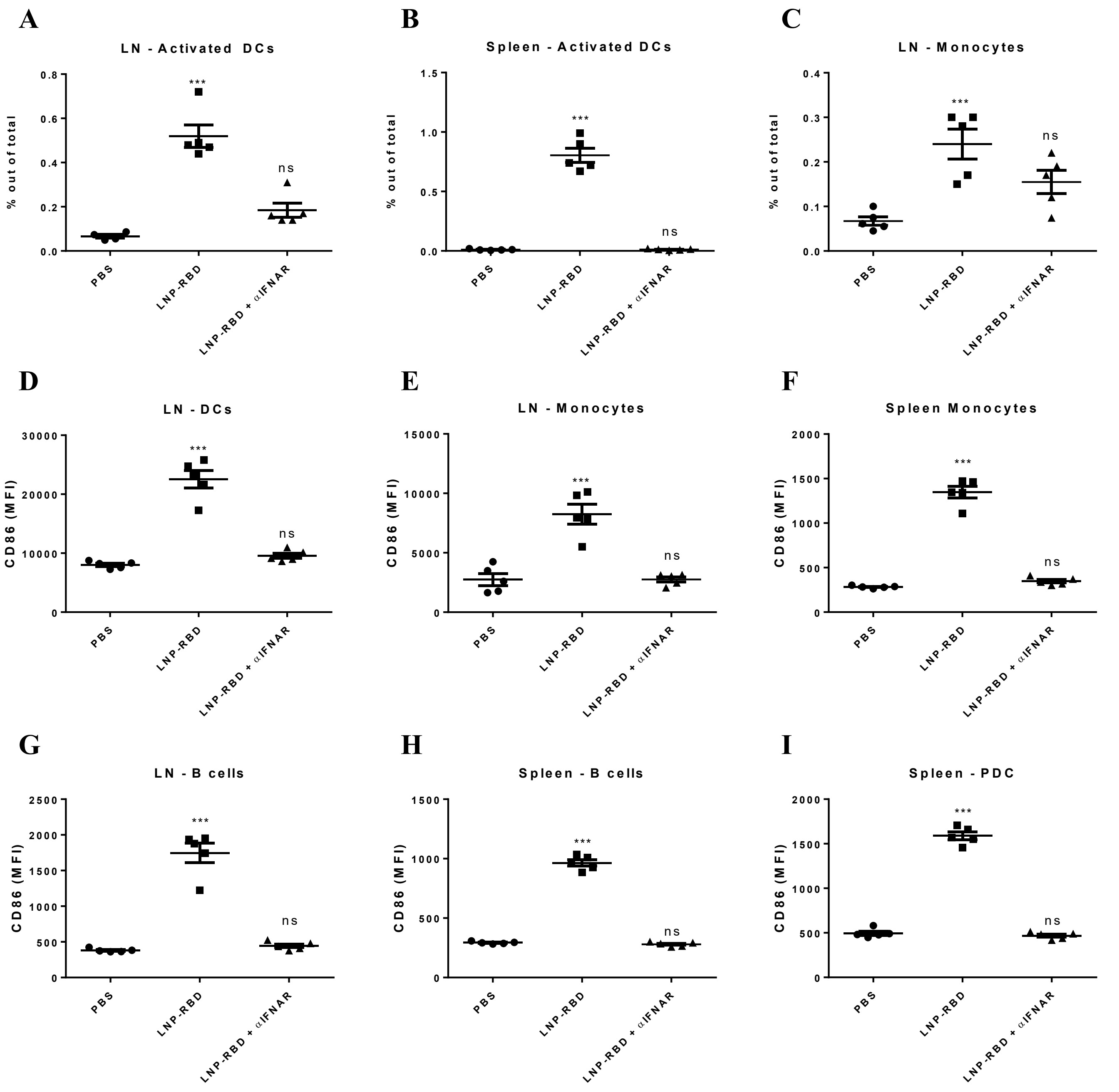

Supplement: Supplementary Figure 1 — Gating strategy to analyze innate immune cell populations in dLNs. Gates of DC subpopulations 1–3 are marked in red. [file DataSheet1.pdf]
